# The Hospital for Curing the Poor in Their Own Homes of Loss of Movement from Paralysis, Rheumatism, and Deformity

**Published:** 1892-12-03

**Authors:** 


### HOSPITAL OPINION.

[Contributions critical, suggestive, controversial, and technical are invited for this column, which is open to everyone who has anything to say that is of practicalvalue and interest.]

### THE HOSPITAL FOR CURING THE POOR IN THEIR OWN HOMES OF LOSS OF MOVEMENT FROM PARALYSIS, RHEUMATISM, AND DEFORMITY.

We have received the following letter, and publish it with Agricola's comments thereon:—

Dear Sir,—Your ideas on the subject of treatment carried on at this hospital do not seem to teem with information. I therefore enclose you a circular showing the object in view. Let me tell you that although it has been started only six months, I have paid for everything without much public aid, and the dances, which seem to trouble you so much, are to put the hospital entirely on its legs, and when that takes place I believe it will be self-supporting. The rooms which I advertised at £2 2s. a week are really to earn money to treat the poor. I think, before you allow any more foolish letters to appear in your columns, it would be better to come round and investigate matters first, and I shall be very happy to show you the hospital and explain my views any day you like at three p.m. I think at the present moment hospitals are doing their best to injure doctors in their private practice by taking paying patients at small fees who can well afford to pay their own medical man. Two cases came before my notice during the seven years I was at a hospital. One was an out-patient, who came up to see me from Brighton. One day she asked me if I could see her sooner, as it was so uncomfortable waiting with the other patients. I said, "You must have discomforts if you come for nothing." "For nothing," she said; "I pay the secretary 10s. every time I come." Another patient was in the hospital, stopping for months at 10s. a week, and after she had gone I found out that she had £600 to £700 a year. Of course, I do not impute anything to the secretary, as he is one of the most honourable men I know; but it is the system which admits people without proper inquiry. And people who are destitute and really in want of treatment are sent from hospital to hospital, although they present letters from subscribers, simply because this system exists of taking paying patients; and, of course, as long as the secretaries of different hospitals can get money from patients the poor may go to the devil. St. Thomas's Hospital charge from £3 3s. upwards a week; the Paralytic Hospital charge from £1 1s. to £4 4s. a week; and I don't think it is out of the way charging from £1 1s. to £2 2s., as I am doing.—I am, yours faithfully,

F. Graham-Bennett.

*** In reference to the foregoing, it may be remarked that no pretence was made to "teem with information" concerning an institution of which we had never heard except through the medium of the advertisement we commented upon. Mr. Graham-Bennett's letter will enable our readers to judge whether we erred in refusing to treat the new-born institution as a serious claimant to the title and dignity of a hospital.—Agricola.

